# Evaluation of the environmental factors influencing the quality of *Astragalus membranaceus* var. *mongholicus* based on HPLC and the Maxent model

**DOI:** 10.1186/s12870-024-05355-3

**Published:** 2024-07-23

**Authors:** Pengbin Dong, Lingjuan Wang, Daiyu Qiu, Wei Liang, Jiali Cheng, Hongyan Wang, Fengxia Guo, Yuan Chen

**Affiliations:** College of Agronomy, College of Life Science and Technology, State Key Laboratory of Aridland Crop Science, Gansu Agricultural University, Lanzhou, 730070 China

**Keywords:** *Astragalus membranaceus* var. *mongholicus*, Quality evaluation, Species distribution

## Abstract

**Background:**

In recent years, global climate change in tandem with increased human activity has resulted in habitat degradation or the migration of rare medicinal plants, potentially impacting the quality of medicinal herbs. *Astragalus membranaceus* var. *mongholicus* is a valuable bulk medicinal material in Northwest China. As the demand for this medicinal herb continues to increase in both domestic and international markets, ensuring the sustainable development of high-quality Astragali Radix is important. In this study, the maximum entropy (Maxent) model was applied, thereby incorporating 136 distribution records, along with 39 environmental factors of *A*. *membranaceus* var. *mongholicus*, to assess the quality zonation and potential distribution of this species in China under climate change.

**Results:**

The results showed that the elevation, annual mean temperature, precipitation of wettest month, solar radiation in June, and mean temperature of warmest quarter were the critical environmental factors influencing the accumulation of astragaloside IV and Astragalus polysaccharide in *A*. *membranaceus* var. *mongholicus*. Among the twelve main environmental variables, annual mean temperature, elevation, precipitation of the wettest month, and solar radiation in November were the four most important factors influencing the distribution of *A*. *membranaceus* var. *mongholicus*. In addition, ecological niche modelling revealed that highly suitable habitats were mainly located in central and western Gansu, eastern Qinghai, northern Shaanxi, southern Ningxia, central Inner Mongolia, central Shanxi, and northern Hebei. However, the future projections under climate change suggested a contraction of these suitable areas, shifting towards northeastern high-latitude and high-elevation mountains.

**Conclusions:**

The findings provide essential insights for developing adaptive strategies for *A*. *membranaceus* var. *mongholicus* cultivation in response to climate change and can inform future research on this species. By considering the identified environmental factors and the potential impacts of the predicted climate changes, we can visualize the regional distribution of high-quality Radix Astragali and develop conservation strategies to protect and restore its suitable habitats.

**Supplementary Information:**

The online version contains supplementary material available at 10.1186/s12870-024-05355-3.

## Introduction

Traditional Chinese medicine (TCM) has a rich history in China, it has been applied for thousands of years for disease prevention and treatment, and it plays a significant role in the Chinese health care system [1]. Currently, its personalized treatment approach and fewer side effects have garnered increasing global attention [2]. Nevertheless, the complexity of TCM, which comprises dozens to hundreds of chemical components, poses significant challenges in quality control [3, 4]. The quality of TCM is not only influenced by genetic characteristics but also influenced by various ecological factors, including climate, light, and soil factors. The active ingredients of medicinal materials are secondary metabolites of medicinal plants, and their synthesis and accumulation are regulated by enzyme-encoding genes along biosynthetic pathways, which vary among populations and are intrinsic determinants of the herbal medicine quality [5]. Furthermore, the ecological environment of the origin location significantly affects both the advancement in herbal medicine and the quantity and composition of its chemical constituents [6]. Chinese medicinal materials of different origins exhibit varying chemical and active ingredient contents due to differences in corresponding growing environment factors (such as the temperature, humidity, light, altitude, and soil factors), thereby influencing the quality and effectiveness of TCM. Consequently, the development of sophisticated analytical methods and the establishment of models linking the chemical content and ecological factors are important for enhancing TCM quality control measures, emphasizing their significance in ensuring the quality and safety of TCM.

Global climate change has posed a serious threat to the quality of Chinese herbal medicines, mainly through the migration or reduction in suitable distribution areas for medicinal plants [7, 8], and this phenomenon may also lead to changes in the content of active ingredients [9, 10]. Employing species distribution records and climate data to create species distribution models has become a prevalent approach for examining the effects of climate change on species habitats [7, 10, 11]. Currently, multiple species distribution modelling algorithms have been widely adopted, among these, the Maxent model is the most widely used due to its superior predictive accuracy [12, 13]. This model aims to not only forecast the spatial distribution patterns of species but also to evaluate the relationships between species and environmental factors (altitude, soil, temperature, and solar radiation) using the jackknife method [14, 15]. In addition, environmental conditions significantly influence the synthesis and accumulation of secondary metabolites in medicinal plants, which are closely linked to species habitat suitability [16–18]. Suitable habitats for the survival of medicinal plants are also considered a prerequisite for the accumulation of secondary metabolites [19]. Many medicinal plants are distributed within highly suitable areas in real-world environments and exhibit relatively high levels of secondary metabolites [8, 19, 20]. However, the detailed mechanism by which global climate change affects the morphological characteristics, phenological period, and curative effects of medicinal plants is not yet fully understood. Nevertheless, such research results are instrumental in establishing a relationship model between the medicinal plant quality and ecological factors, providing a sound basis for evaluating appropriate cultivation regions for high-quality medicinal herbs and predicting their future geographic distribution.

*A*. *membranaceus* var. *mongholicus* is a perennial herb plant in the Leguminosae family and an important traditional Chinese medicinal material, and its dry roots are the primary source of Radix Astragali [21]. Its dried roots are known for their ability to reinforce vital energy, promote surface cooling and stop sweating, facilitate diuresis, and prevent platoon poisoning [22, 23]. In TCM, Radix Astragali is prescribed for diverse ailments, such as diabetes, hepatitis, bronchitis, and gastric cancer [24]. Modern pharmacological research has shown that among the various chemical components of Huangqi herbs, astragaloside IV, Astragalus polysaccharides, calycosin-7-glucoside, and total flavonoids exhibit multiple pharmacological effects, including anti-viral myocarditis, antitumour properties, and other functions [17]. Moreover, Astragali Radix plays a pivotal role in the health product, food, and cosmetics sectors, as its long-term usage enhances the human immune system and promotes rejuvenation [25]. In recent years, the increasing demand for Astragali Radix in both domestic and international markets, combined with the repercussions of climate change and human activities, has posed a serious threat to wild populations. Consequently, *A*. *membranaceus* var. *mongholicus* has been designated as endangered by the “China Rare Endangered Plant Directory”[26]. At present, Astragali Radix is mainly cultivated in Gansu, Inner Mongolia, Shanxi, and Shaanxi provinces in China, which are the authentic producing areas of this variety. Most of these regions exhibit a temperate monsoon climate, but differ significantly in factors such as altitude, precipitation, diurnal temperature range, and solar radiation. It should be determined whether differences in terrain, climate, and other ecological factors could lead to variations in the quality of this medicinal plant.

**Fig. 1:**
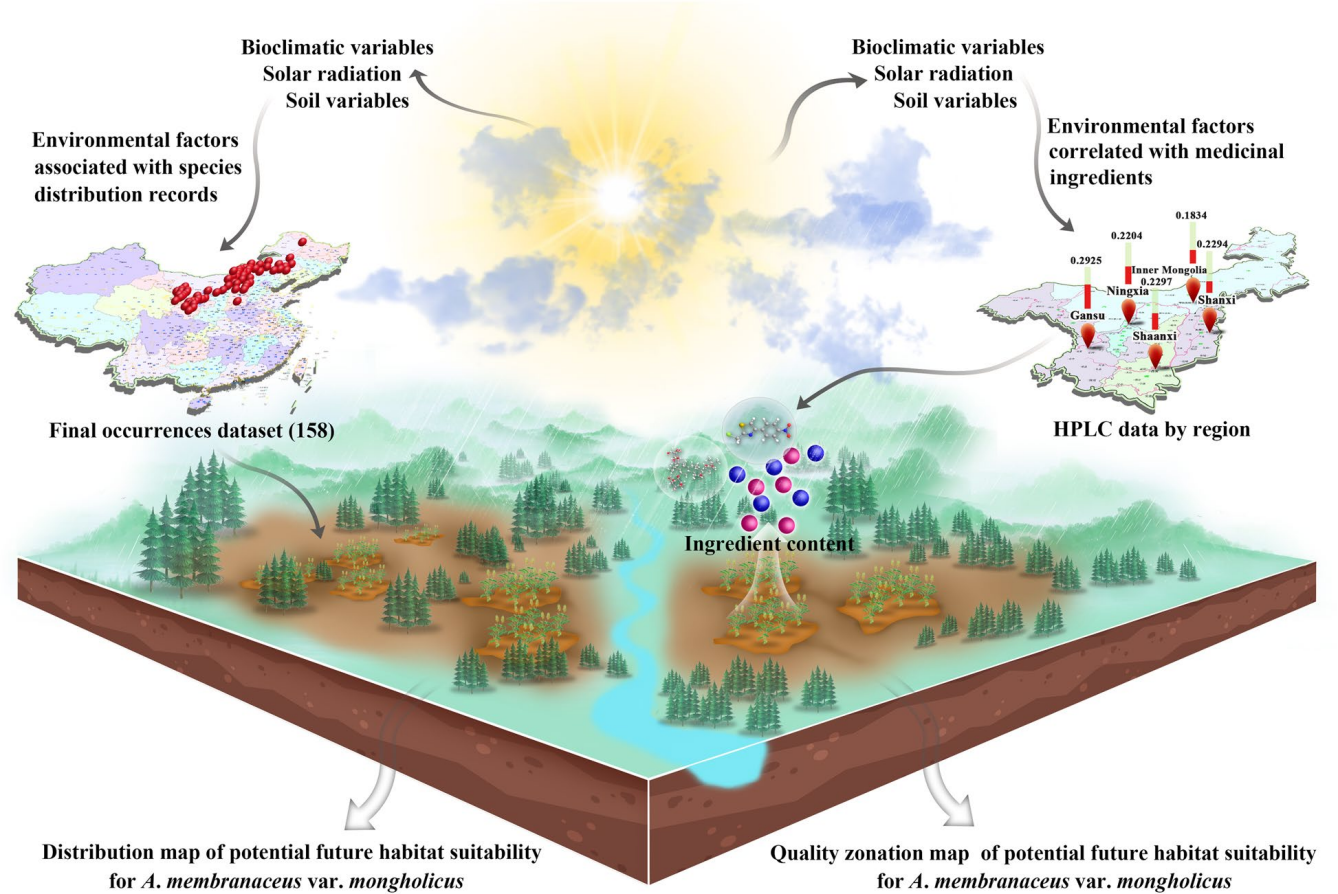
Schematic illustration of the assessment strategy for the impact of ecological factors on the ecological suitability and quality of *A*.* membranaceus* var. *mongholicus*

Climatic factors influence not only the suitable cultivation areas for medicinal plants but also the formation and accumulation of chemical substances. In this study, we quantified the contents of astragaloside IV, and calycosin-7-glucoside in *A*. *membranaceus* var. *mongholicus* samples of different origins using HPLC. A Maxent model was established by integrating occurrence and environmental variable data. Based on HPLC and the Maxent model, the correlation between chemical components and ecological factors was further explored through spatial analysis and the mapping function of GIS software combined with multivariate statistical analysis. The obtained results provide a reasonable basis for the assessment of habitat suitability, sustainable cultivation, and resource conservation for endangered medicinal plants. Fig. 1 shows a schematic representation of the proposed methodology.

## Results

### Chemical analysis of the component content by HPLC

The active ingredient content in *A. membranaceus* var. *mongholicus* was determined by HPLC, according to the content determination protocol outlined in the Chinese Pharmacopoeia 2020 edition (Table S1). To explore the relationships between the chemical components of this species and various ecological factors, bivariate correlation analysis was performed. Astragaloside IV and Astragalus polysaccharide levels, derived from samples obtained in different regions, served as the dependent variables (Y1, and Y2, respectively), with the ecological factors identified by the Maxent model as the independent variables (X1, X2…, X4). Stepwise regression analysis was conducted to establish a regression equation for the astragaloside IV and Astragalus polysaccharide contents and their respective environmental factors: Y_1_ = 0.803–0.626X_1_-0.341X_2_ + 0.224X_3_-0.195X_4_-0.217X_5_ (X_1_ is the mean diurnal range; X_2_ represents the elevation; X_3_ is the annual precipitation; X_4_ is the mean temperature of coldest quarter; and X_5_ is the solar radiation in June); Y_2_ = 27.427 + 0.961X_1_-0.163X_2_-0.573X_3_-1.832X_4_ + 1.567X_5_-0.028X_6_ (X_1_ denotes the annual mean temperature; X_2_ denotes the precipitation of wettest month; X_3_ is the mean temperature of warmest quarter; X_4_ is the precipitation of wettest quarter; X_5_ denotes the precipitation of warmest quarter; and X_6_ represents min temperature of coldest month). Statistical analysis using the F and T tests confirmed the validity of the above regression equations and their respective Pearson correlation coefficients (*P* < 0.05). This suggests the possibility of leveraging these regression equations to predict the chemical content of *A*. *membranaceus* var. *mongholicus* based on the ecological environment.

### Correlation of the chemical composition with environmental variables

**Fig. 2:**
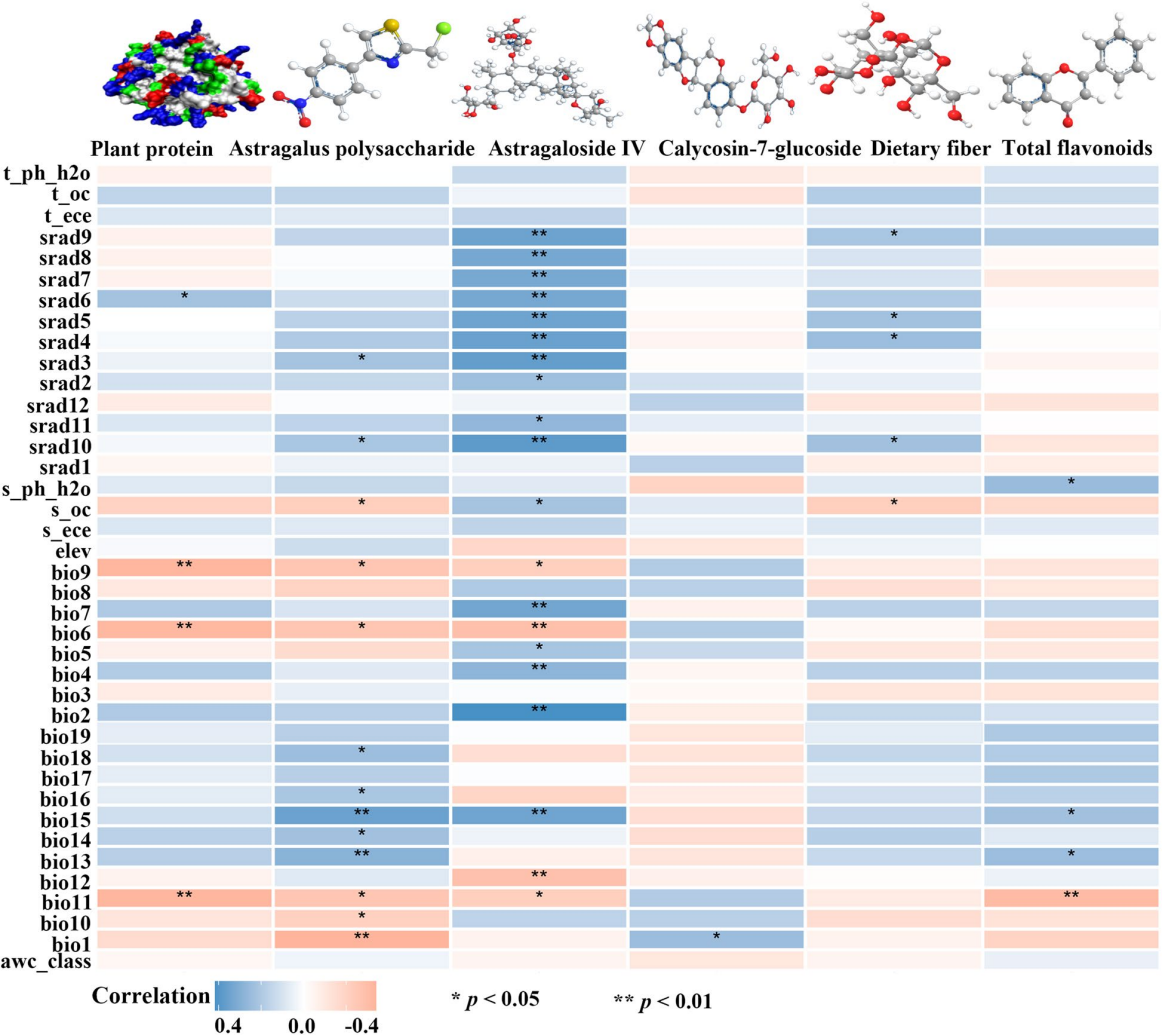
Geographical distribution of *A*.* membranaceus* var. *mongholicus*. Note: red points represent species distribution records

Pearson correlation analysis revealed that the contents of astragaloside IV, Astragalus polysaccharide, and total flavonoids were significantly correlated with environmental factors (Fig. 2). The content of Astragalus polysaccharide was significantly correlated with temperature factors (Bio1, Bio5, Bio8, and Bio10) and precipitation factors (Bio12, Bio13, Bio16, and Bio18). Similarly, the total flavonoid content was significantly associated with precipitation factors Bio12, Bio13, Bio16, and Bio18. In addition, astragaloside IV content was highly correlated with soil variables (t_oc), solar radiation variables (srad1, srad3, srad4, srad5, srad6, srad9, srad10, and srad11), elevation, temperature factors (Bio2, Bio5, Bio6, Bio7, Bio8, and Bio10,) and precipitation factor (Bio15).

### Current potential distribution and quality zonation estimates

**Fig. 3:**
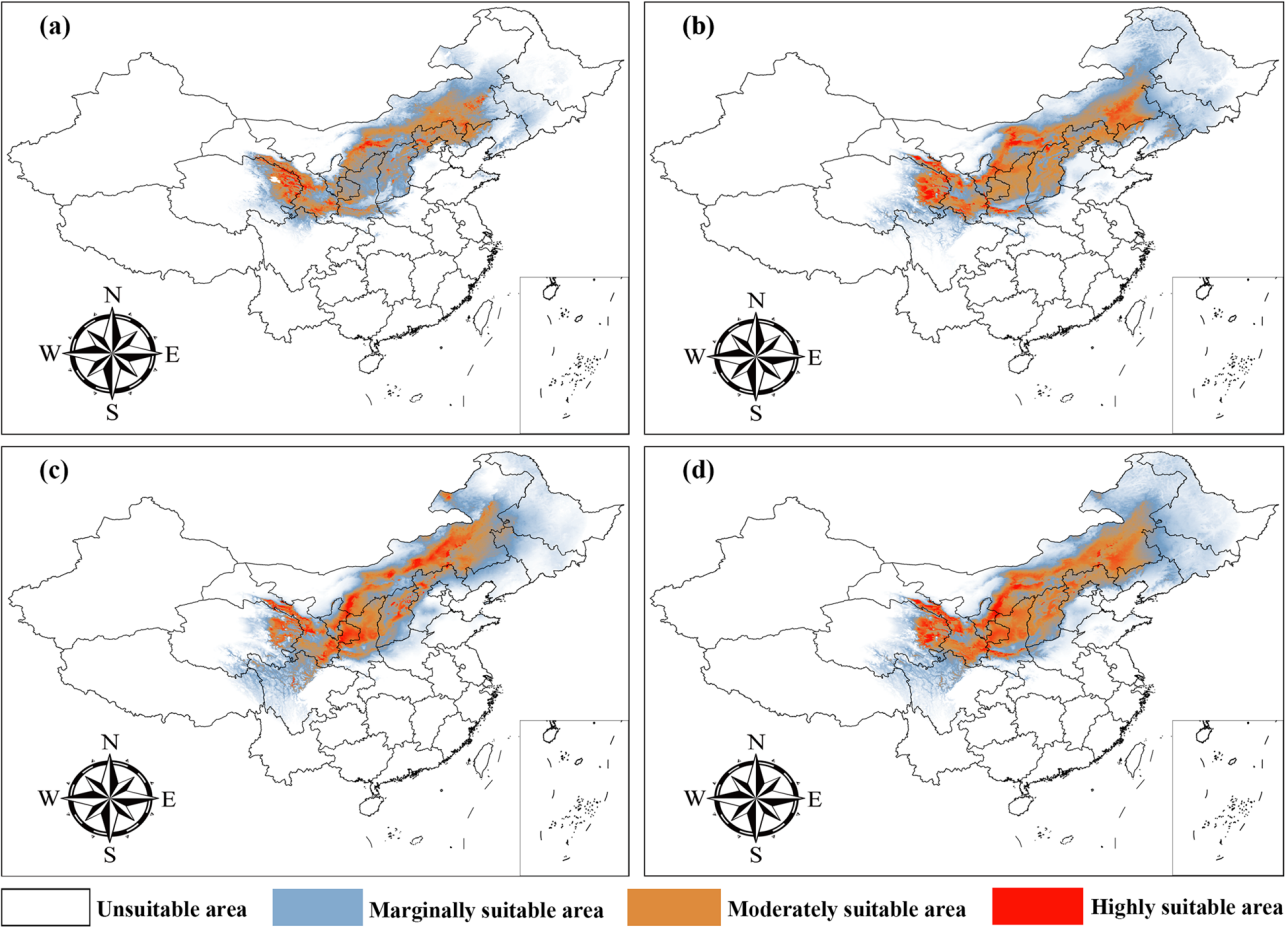
Correlation analysis of environmental variables. Blue represents positive correlations and orange represents negative correlations

Under the current climate scenario, the ecological niche model suggested that the suitable habitats for *A*. *membranaceus* var. *mongholicus* are primarily located in the northern and northeastern regions of China, exhibiting a zonal distribution (Fig. 3a). The low-suitability zones covered an area of 80.07 × 10^4^ km^2^, the medium-suitability zones covered an area of 70.38 × 10^4^ km^2^, the high-suitability zones covered an area of 41.85 × 10^4^ km^2^, and the total suitable habitat area reached approximately 192.29 × 10^4^ km^2^, accounting for 20.04% of the total territory of China (Table 1). The core distribution area predicted by the model is consistent with the current distribution records of this species, mainly encompassing the semiarid regions of China, including western and central Gansu, eastern Qinghai, northern Shaanxi, southern Ningxia, central Inner Mongolia, central Shanxi, and northern Hebei (Fig. 3a). Figure 3b and c show the spatial distributions of astragaloside IV and Astragalus polysaccharides, respectively. By merging these two spatial distributions with a habitat suitability map, we constructed a quality zoning map for *A*. *membranaceus* var. *mongholicus* in China (Fig. 3d). Moreover, the distribution pattern of the medicinal components in *Astragalus membranaceus* var. *mongholicus* revealed that the key active compounds were primarily located in central Gansu, eastern Qinghai, northern Shaanxi, Shanxi, central Inner Mongolia, and northern Hebei (Fig. 3d). Specifically, the regions with high, medium, and low suitability measures covered areas of 70.20 × 10^4^ km^2^, 76.84 × 10^4^ km^2^, and 141.56 × 10^4^ km^2^, respectively (Table 1).

**Table 1:** *A*. *membranaceus* var. *mongholicus* potential distribution and quality zones areas

Types	Period	Area of each suitable region (× 10^4^ Km^2^)			
		Marginally suitable region	Moderately suitable region	Highly suitable region	Total sutable region
*A*. *membranaceus* var.* mongholicus*	Current	80.06597	70.38021	41.84722	192.29340
Astragaloside IV	Current	127.7951	69.08681	60.12674	257.00865
Astragalus polysaccharides	Current	96.23959	81.96875	52.98959	231.19793
Quality zoning map	Current	141.55900	76.83854	70.20313	288.60067

### Suitable distribution and quality zonation under future climate scenarios

**Fig. 4:**
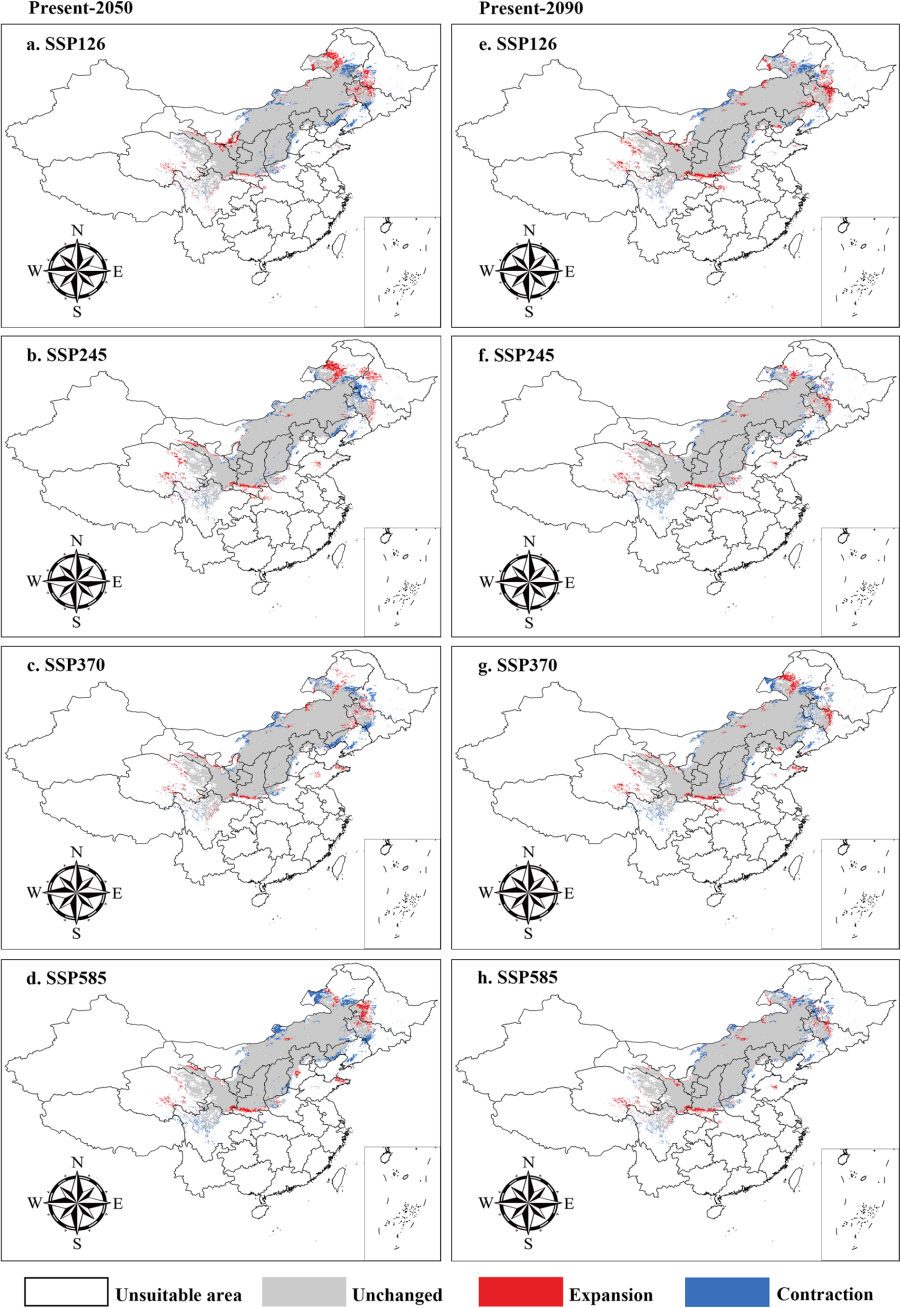
Correlation analysis between the content of six components of *A*.* membranaceus* var. *mongholicus *with environmental factors

The spatial distribution and medicinal quality distribution patterns of *A*. *membranaceus* var. *mongholicus* under different climate change scenarios (SSP126, SSP245, SSP370, and SSP585) in the 2050s and 2090s were predicted based on three high-resolution general circulation models (GCMs). The dynamic changes in the suitable habitat and medicinal quality spatial distribution of the species are shown in Fig. 4, Fig. S1-5, and Table 2 under these scenarios. Compared to that under the current climate scenario, under the SSP126 scenario, the total suitable area is expected to increase (Fig. 4a, and e and Table 2). Conversely, under SSP370 and SSP585, the suitable area decreased, and the core distribution significantly decreased, especially under the SSP585 scenario (Fig. 4d, and h and Table 2). Furthermore, in terms of quality zoning, the spatial distribution of *A*. *membranaceus* var. *mongholicus* fluctuated with climate change. Overall, the species response to climate change was consistent, with the total quality distribution area contracting during all periods except for expansion under the SSP126-2090s climate scenario (Fig. S3-5 and Table S2).

**Table 2:** The potential distribution area of *A*. *membranaceus* var. *mongholicus* in the 2050s and 2090s

Species	Model	Period	Area of each suitable region (× 10^4^ Km^2^)			
			Unsuitable region	Unchanged region	Expansion region	Contractionregion
*Astragalus membranaceus* var. *mongholicus*	BCC-CSM2-MR	Present **vs** SSP126-2050s	659.94	25.78	15.19	16.12
		Present **vs** SSP245-2050s	662.33	25.43	12.80	19.53
		Present **vs** SSP370-2050s	671.65	23.05	3.49	43.42
		Present **vs** SSP585-2050s	667.51	24.67	7.62	27.18
		Present **vs** SSP126-2090s	666.73	24.97	17.41	12.03
		Present **vs** SSP245-2090s	668.54	25.03	6.60	23.58
		Present **vs** SSP370-2090s	657.73	26.18	8.40	24.15
		Present **vs** SSP585-2090s	664.83	24.95	10.31	24.38
	BCC-CSM1.1	Present **vs** RCP2.6-2050s	654.52	25.38	20.48	20.06
		Present **vs** RCP4.5-2050s	654.29	25.12	20.71	22.68
		Present **vs** RCP6.0-2050s	658.59	25.39	16.41	19.95
		Present vs RCP8.5-2050s	655.81	25.34	19.19	20.49
		Present vs RCP2.6-2090s	662.76	24.14	12.24	32.48
		Present vs RCP4.5-2090s	660.07	24.45	14.94	29.41
		Present vs RCP6.0-2090s	659.98	22.83	15.02	45.55
		Present vs RCP8.5-2090s	660.94	23.51	14.06	38.70
	MIROC5	Present **vs** RCP2.6-2050s	662.74	24.93	12.26	24.62
		Present **vs** RCP4.5-2050s	663.37	24.23	11.64	31.55
		Present **vs** RCP6.0-2050s	666.02	23.83	8.98	35.54
		Present vs RCP8.5-2050s	666.88	23.31	8.13	40.81
		Present vs RCP2.6-2090s	664.69	24.21	10.30	31.78
		Present vs RCP4.5-2090s	665.69	24.22	9.31	31.71
		Present vs RCP6.0-2090s	662.99	24.35	12.01	30.38
		Present vs RCP8.5-2090s	662.43	24.19	12.58	31.98

### Changes in the geometric centre of *A*. *membranaceus* var. *mongholicus* in the suitable areas under future climate conditions

**Fig. 5:**
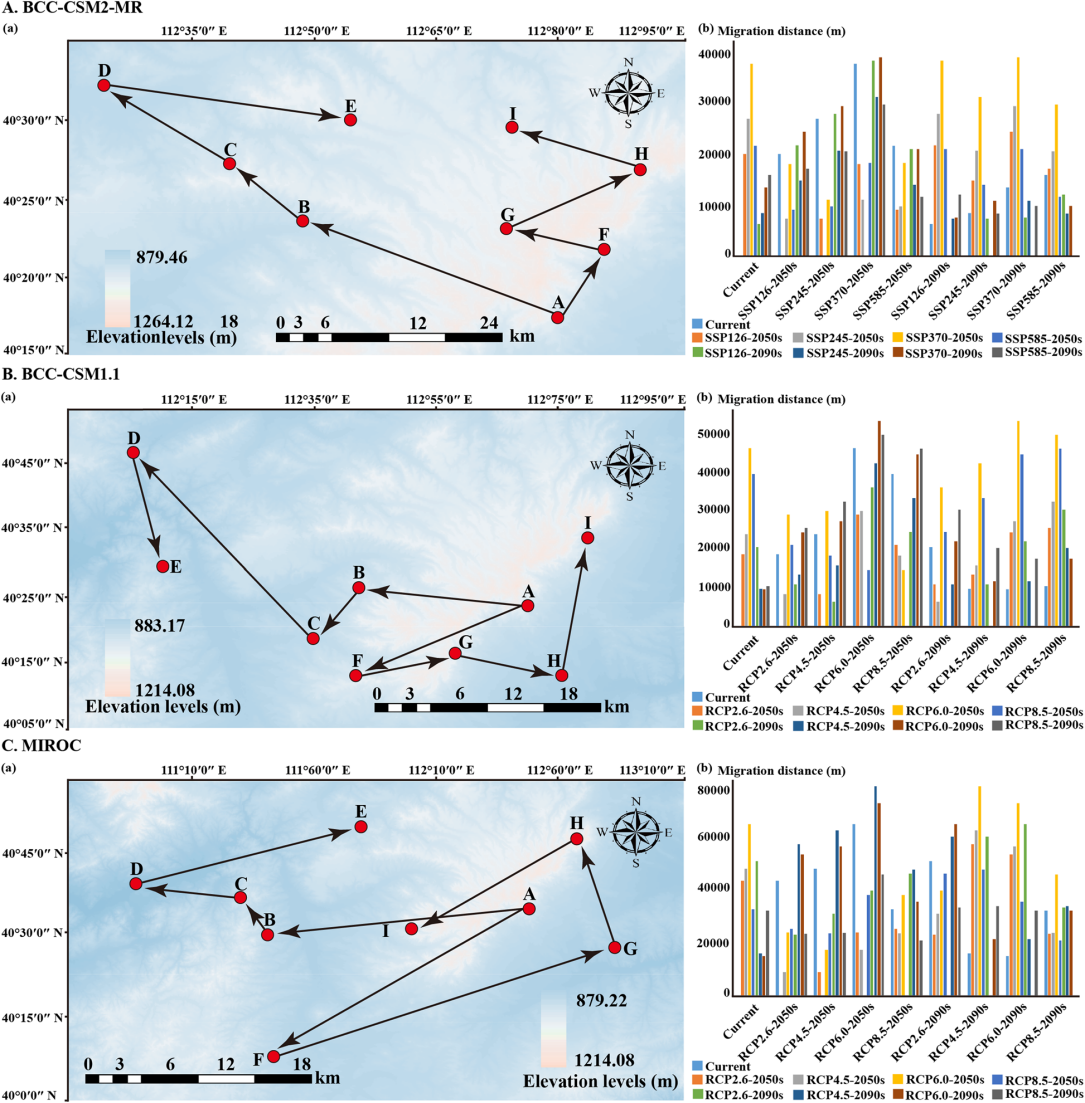
Potential distribution and quality zones areas for *A*.* membranaceus* var. *mongholicus* by the Maxent model based on optimized parameters. **a** potential geographical distribution of *A*.* membranaceus* var. *mongholicus*; **b** spatial distribution of astragaloside IV content; **c** patial distribution of astragalus polysaccharide content; **d** The quality zoning map of *A*.* membranaceus* var. *mongholicus. *White, blue, orange and red areas represent not suitable, marginally suitable, moderately suitable, and highly suitable areas, respectively

In this study, the geometric center defines the central point of the potentially suitable area, which represents the location and migration trajectory of the suitable habitats and the quality zonation for the suitable habitats of *A*. *membranaceus* var. *mongholicus*. The findings derived from the general circulation models indicate that the direction and distance between the centroids of the suitable habitats for *A*. *membranaceus* var. *mongholicus* and the suitable habitats for quality zonation differ under the different climate scenarios compared to those during the current period (Fig. 5 and Fig. S6). Currently, the geometric centres of the suitable habitats for *A*. *membranaceus* var. *mongholicus* and the suitable habitats for herb quality zonation (astragaloside IV and Astragalus polysaccharide) are located in Caonian Manzu township, Liangcheng County, Ulaanchabu city, Inner Mongolia Autonomous Region (112.746377 E, 40.191868 N), and Chengguan town, Xinghe County, Ulaanchabu city (113.88399 E, 40.910892 N). In the 2050s and 2090s, the centroid of the suitable habitats for *A*. *membranaceus* var. *mongholicus* shifted northwards compared to that in this study (Fig. 5A, B, and C). The BCC-CSM2-MR model indicates that by the 2050s, the centroids of the suitable areas for this species under the SSP 126, SSP 245, SSP 370, and SSP 585 climate scenarios are predicted to all move to northwestern Liangcheng County, Ulaanchabu city, and the Inner Mongolia Autonomous Region, with migration distances of 17.88, 24.05, 33.73, and 19.34 km, respectively (Fig. 5A). By the 2090s, the centroids of the suitable areas for *A*. *membranaceus* var. *mongholicus* are expected to migrate further northwards and reach Caonian Manzu township, Guanghanying township, Tiancheng Township, Liangcheng County, and Ulaanchabu city, with migration distances of 5.50, 7.49, 11.95, and 14.17 km under SSP126, SSP245, SSP370, and SSP585, respectively (Fig. 5A). Moreover, the prediction results of the BCC-CSM1.1 and MIROC5 models revealed that the migration direction of the centroid of the suitable areas for *A*. *membranaceus* var. *mongholicus* was similar to that of the BCC-CSM2-MR model (Fig. 5B, C). Similarly, the predictions of the herb quality (astragaloside IV and Astragalus polysaccharide) in the 2050s and 2090s indicated a migration trend consistent with that of the suitable habitats for *A*. *membranaceus* var. *mongholicus* species (Fig. S6A, B, C). According to the BCC-CSM2-MR model by the 2050s, the SSP126, SSP245, SSP370, and SSP585 climate scenarios will result in migration distances of 28.75, 23.05, 77.15, and 38.40 km, respectively (Fig. S6A). By the 2090s, the centroids are expected to continue to shift to the northeast, with distances of 12.08, 18.50, 46.39, and 28.53 km, respectively (Fig. S6A). In addition, the predicted spatial distributions of the BCC-CSM1.1 and MIROC5 models suggest that the centroid of the suitable areas is expected to shift to the northwest and northeast at two distinct time horizons (2050 and 2090, respectively) (Fig. S6B, C).

### Environmental variables affecting the distribution of species

By integrating the contributions of the nine selected environmental variables to the model with results of the jackknife method, we identified the key climatic factors. As indicated in Table S3, in this study, among the twelve environmental variables, the main determinants affecting the distribution of *A*. *membranaceus* var. *mongholicus* were Bio13 (precipitation of wettest month), Elev (elevation), Srad6 (solar radiation in June), Srad11 (solar radiation in December), S_ph_h2o (topsoil pH (H_2_O)), Bio1 (annual mean temperature) and Bio18 (precipitation of warmest quarter). The main climatic factors influenced the potential geographical distribution of this species, and the contribution rate and permutation importance were 95.6% and 94.5%, respectively. Based on the response curves of the main climate factors of *A*. *membranaceus* var. *mongholicus*, the ranges for these six environmental variables were as follows: Bio13 (71.40–128.52 mm), Elev (792.47–2,177.56 m), Srad6 (22,857.14–24,142.88 kJm^−2^day^−1^), Srad11 (8,840.00–10,800.00 kJm^−2^day^−1^), S_ph_h2o (5.50–8.00 − log (H +)), and Bio1 (3.05–7.50 ◦C) (Fig. S7).

## Discussion

### Influence of the environmental factors on the habitat of this species

Climate change notably impacts the survival, distribution and quality of medicinal plants, and changes in the geographical distribution patterns of medicinal plants usually reflect the dynamic characteristics of climate [27]. Kumar et al. suggested that the species distribution patterns of many medicinal plants are mainly influenced by precipitation, temperature, and topographic factors [28]. In this study, we utilized the jackknife test in the Maxent model to assess the bioclimatic variables influencing the geographical and medicinal quality distribution patterns of *A*. *membranaceus* var. *mongholicus.* The analysis results for the current climate revealed that elevation, annual mean temperature, solar radiation in June, solar radiation in December, and substrate-soil pH (H_2_O) were the main factors influencing the potential geographical distribution of *A*. *membranaceus* var. *mongholicus*, with elevation yielding the greatest contribution. Over the twentieth century, the massive emissions of greenhouse gases caused the global average surface temperature to rise by 1.8 ℃ [29], which has exerted a significant impact on the annual mean temperature and solar radiation [30]. In certain areas of the middle temperate zone or warm temperate zone of China, the summers are hot and rainy, while the winters are cold and dry [31]. The distribution of *A*. *membranaceus* var. *mongholicus* requires specific conditions, such as elevation (792.47 to 2,177.56 m), solar radiation in June (22,857.14 to 24,142.88 kJm^−2^day^−1^), solar radiation in December (8,840.00 to 10,800.00 kJm^−2^day^−1^), annual mean temperature (3.05 to 7.50 ℃), and S_ph_h2o (5.50–8.00 − log (H +)) (Fig. S7). These specific needs hinder its expansion south of the Qinling Mountains due to constraints related to elevation, solar radiation, and temperature. This is the reason why this species is mainly distributed in plateau grasslands and areas with sufficient sunlight and moderate precipitation but is not distributed or planted in hot and humid areas in the south. Consistent with prior studies, our findings reinforce that *A*. *membranaceus* var. *mongholicus* is adept at withstanding cold and drought but struggles in intensely hot or waterlogged environments [32].

In this study, we overlaid spatial distribution maps of the active ingredients astragaloside IV and Astragalus polysaccharides onto a habitat suitability distribution map to produce a zoning map of the quality of Astragali Radix in China (Fig. 3d). High-quality *A*. *membranaceus* var. *mongholicus* was primarily distributed in central and western Gansu, eastern Qinghai, central Ningxia, northern Shaanxi, northern Hebei, central Inner Mongolia, and Shanxi, and its distribution pattern was influenced by the altitude and mean annual temperature (Fig. 3d). Ecological factors are among the key factors influencing the quality of herbs [25], among which altitude imposes a certain influence on the accumulation of ingredients in authentic herbs [33]. High-quality Astragali Radix primarily thrives in mountainous or semimountainous regions with altitudes varying between 800 and 1,300 m, while lower-altitude growing areas are prone to disease due to heat and humidity and limited air mobility [34]. In addition, a comparison between the herbal medicine quality distribution map and the habitat suitability distribution map revealed that the zones of a lower herbal medicine quality precisely coincided with the areas of low or unsuitable habitats. These results suggest that environmental variables that may be detrimental to the growth and development of *A*. *membranaceus* var. *mongholicus* are also unfavourable for the accumulation of its active components. However, it is crucial to recognize that a suitable habitat is not always a high-quality production area for medicinal materials. The synthesis and accumulation of plant secondary metabolites are greatly influenced by their inherent physiological and biochemical processes [5]. Many active ingredients of medicinal plants are secondary metabolites, which are the result of plant adaptation and selection to complex external environmental variations throughout their evolutionary process [35, 36]. Experiments focused on determining the effect of environmental stress on medicinal plants have shown that adverse conditions may stimulate the accumulation and release of plant secondary metabolites [37, 38]. Huang Luqi et al. clearly suggested that adversity may promote the formation of authentic medicinal herbs, possibly resulting in their authentic product areas becoming spatially distributed at the edge of their entire species range [39]. This theory elucidates the differences in the main ecological variables between the geographical distribution pattern of *A*. *membranaceus* var. *mongholicus* and the distribution of the Astragali Radix quality in this study.

### Changing spatial patterns of this species under future climate scenarios

Under future climate conditions, the results of three high-resolution GCMs (BCC-CSM1.1, BCC-CSM2-MR and MIROC5) indicated that the suitable areas for *A*. *membranaceus* var. *mongholicus* will decrease under three distinct climate scenarios (SSP126, SSP370, and SSP585) in the 2050s and 2090s. Similar phenomena have been observed for other medicinal plants, such as *Paeonia rockii* and *Sinopodophyllum hexandrum* [27, 40]. Over the past century, the global mean temperature has increased by 0.74 ℃, and it is expected that the surface temperature will continue to rise above 2.2 ℃ in the 2090s [41]. Consequently, the suitable habitats and herb quality zones for *A*. *membranaceus* var. *mongholicus* will generally decrease, and fragmentation will be reduced. Under the SSP126-2090s emission scenario, the suitable distribution range of *A*. *membranaceus* var. *mongholicus* significantly increased, particularly in eastern Inner Mongolia, western Heilongjiang and western Jilin, which is generally consistent with the northward migration trend of its common medicinal plant *Codonopsis pilosula* in mixed forests (Fig. 4) [42]. Under the SSP585-2090s emission scenario, the suitable habitat distribution of *A*. *membranaceus* var. *mongholicus* was particularly concentrated in the central region of Inner Mongolia, northern areas of Hebei, northern parts of Shanxi, and central region of Gansu (Fig. 4). Furthermore, a comparison of the topographical characteristics revealed that *A*. *membranaceus* var. *mongholicus* will migrate to higher latitudes and altitudes under future climate conditions (Fig. 5A, B, C). Nevertheless, changes in the elevational distribution of species may involve biological or other disturbance factors in addition to climatic influences [43]. Here, two plausible reasons may explain why the suitable distribution areas of this species will migrate to higher latitudes and elevations in the future. First, climate warming is the main factor affecting the altitudinal migration of species. This is because altitude is associated with many factors that directly affect the phenology of organisms, especially climatic factors, such as mean annual temperature and seasonal temperature variations, which may directly influence key processes that determine the dynamics of species distributions [44]. Pauli et al. showed that in studies of vascular plant diversity in major European mountain ranges, past warming has caused most plants to migrate upwards [45]. Second, anthropogenic disturbances (e.g., deforestation, grazing, farming, and tourism) can significantly affect the elevational distribution of species [46, 47]. In lower-elevation regions, especially in densely populated areas (e.g., Europe), anthropogenic factors may also cause species to migrate towards higher altitudes [48, 49]. In recent decades, the tourism industry has flourished in China, with an increase in tourism and recreational activities in the Inner Mongolian Plateau and Loess Plateau regions, leading to the expansion of tourism infrastructure (hotels, scenic spots, and transport). Therefore, we speculate that this may be one of the reasons for the migration of this species to higher latitudes and elevations.

### Relationship between the chemical composition and quality zonation of *A*. *membranaceus* var. *mongholicus*

Correlation analysis between the active ingredients of *A*. *membranaceus* var. *mongholicus* and environmental variables revealed that the Astragalus polysaccharide content was mainly influenced by temperature and precipitation, whereas the accumulation of astragaloside IV was primarily affected by soil variables, solar radiation variables, altitude, temperature and precipitation (Fig. 2). Compared with the accumulation and synthesis of Astragalus polysaccharides, the accumulation and synthesis of astragaloside IV in this species are more easily affected by the ecological environment. Astragaloside IV is not only the primary bioactive saponin found in *A*. *membranaceus* var. *mongholicus* but also a major component of commercial saponin extracts [50, 51]. Therefore, high-quality herbs can be cultivated through artificial control of environmental factors in the cultivation area and the implementation of sound field management practices, while large quantities of Astragalus methyloside and Astragalus polysaccharide can be obtained.

In this study, the regional suitability of high-quality *A*. *membranaceus* var. *mongholicus* indicated that the most suitable areas for its cultivation were western Gansu, eastern Sichuan, central Inner Mongolia and northern Shanxi. These areas exhibit a distinct topography and climate characterized by well-defined seasons, low precipitation, and significant temperature fluctuations that are favourable for the accumulation of secondary metabolites [25]. The soil requirements of this species are not highly specific, but the texture and thickness of the soil layer can impact its yield and quality [52]. The findings of this study provide additional evidence indicating that the optimal distribution areas mainly encompass elevated terrains characterized by effective drainage systems.

## Conclusion

In this study, HPLC and the Maxent model were employed to investigate the relationships between the spatial distribution of *A*. *membranaceus* var. *mongholicus* and ecological factors, and the changes in the medicinal quality under future climate scenarios were assessed. The results showed that the elevation, annual mean temperature, solar radiation in November, solar radiation in June, and precipitation in the wettest month were critical environmental factors for the accumulation of astragaloside IV and astragalus polysaccharide. Suitable habitats for* A*. *membranaceus* var. *mongholicus* were mainly located in the Hexi Corridor and central Gansu, eastern Qinghai, northern Shaanxi, southern Ningxia, central Inner Mongolia, central Shanxi, and northern Hebei. The future climate change scenarios suggested a reduction and a northeastward shift in the suitable distribution areas to higher latitudes and elevations. These findings provide essential insights for developing adaptive strategies for *A*. *membranaceus* var. *mongholicus* cultivation in response to climate change and suggest new approaches for optimizing its cultivation in China.

## Materials and methods

### Plant material and species distribution data sources

Species distribution data for *A*. *membranaceus* var. *mongholicus* were collected through field surveys in its primary growing regions in China between 2019 and 2022. We gathered 39 sample sets from distinct locations: Dingxi city (13), Longnan city (2) in Gansu Province, Yulin city (3) in Shaanxi Province, Datong city (5), Shuozhou city (3) in Shanxi Province, Guyuan city (4) in Ningxia, Hohhot city (3), Baotou city (3), Chifeng city (2) and Bayannur city (1) in Inner Mongolia. These samples were identified by Professor Chen Yuan at Gansu Agricultural University. The dried tissue samples and voucher specimens (No. PRLML2023022) were deposited at the Gansu Agricultural University Herbarium (GAUF). The fresh samples were naturally dried at room temperature (10–15 ℃) at a cool location to ensure a moisture content lower than 10.0% in the dried samples. Then, the samples were finely ground using a pulverizer and passed through a 50-mesh sieve. Finally, the resulting powder from each sample was stored in Ziploc bags for HPLC analysis.

**Fig. 6:**
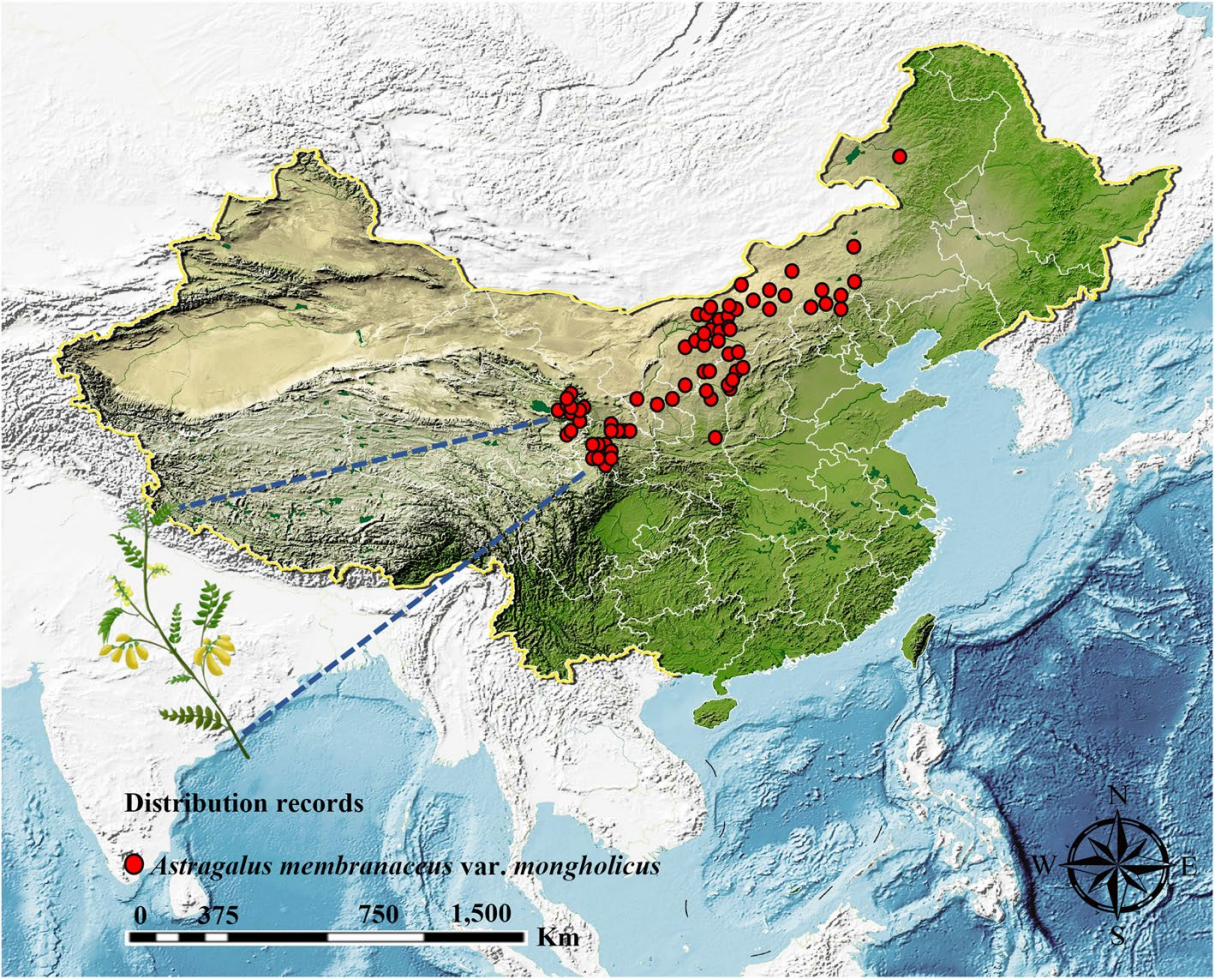
Spatial changes of *A*.* membranaceus* var. *mongholicus *in China under emission scenarios of the 2050s and 2090s. White, Gray, Red and Blue areas represent not suitable, unchanged suitable, expansion suitable, and contraction suitable areas, respectively. **a**-**d**, the 2050s; **e**-**h**, the 2090s; **a**, **e**, future climate scenario SSP126; **b**, **f**, future climate scenario SSP245; **c**, **g**, future climate scenario SSP370; **d**, **h**, future climate scenario SSP585. (Note: general circulation model BCC-CSM2-MR)

Furthermore, we reviewed the literature and consulted the Chinese Virtual Herbarium (https://www.cvh.ac.cn/), Global Biodiversity Information Facility database (http://www.gbif.org), and Teaching Specimen Resource Sharing Platform (http://mnh.scu.edu.cn/) to obtain 185 distribution points of *A*. *membranaceus* var. *mongholicus*. To prevent spatial autocorrelation of the species data from affecting the model prediction results, we performed spatial filtering of the species distribution points by selecting only one occurrence record for each 2 km × 2 km grid. As a result, we obtained 136 effective occurrence records for model calculations (Fig. 6 and Table S4).

### Chemical composition analysis

The contents of protein, total flavonoids, and dietary fibre were determined in accordance with standards GB 5009.3–2016, SZDB/Z 349–2019, and GB 5009.88–2014, respectively. The content of Astragalus polysaccharides was determined using the anthrone-sulfuric acid method. The concentrations of astragaloside IV and calycosin-7-glucoside in the samples were analysed using HPLC. The following adopted chromatographic conditions were used for astragaloside IV: Zorbax Eclipse XDB C18 column (4.6 mm × 250 mm, 5 μm, PN#990,967.902) (Agilent, Palo Alto, CA) protected by a Waters Delta-Pak C18 guard column (Waters Technologies Ireland, Ltd., Wexford, Ireland) and set to a temperature of 20 °C. The mobile phase used for the separation consisted of solvent A (water, deionized) and solvent B (acetonitrile). The elution profile was as follows: 0 → 30 min, 20% → 58% B; 30 → 31 min, 58% → 90% B; 31 → 35 min, 90% B (washing out); 36 → 37 min, 90% → 20% B; 37 → 40 min, 20% B (reconditioning). The flow rate was 1.6 mL/min, the injection volume was 10 μL, and the detection wavelength was 203 nm. Chromatographic conditions under calcein-7-glucoside: XSelectHSST3 C18 column (4.6 mm × 250 mm, 5 μm) and set to a temperature of 35 °C. The mobile phase used for the separation consisted of solvent A (acetonitrile) and solvent B (phosphoric acid). The elution profile was as follows: 0 → 5 min, 10% → 35% A, 90% → 65% B; 5 → 10 min, 35% → 65% A, 65% → 35% B; 10 → 13 min, 65% → 85% A, 35% → 15% B; 13 → 15 min, 85% A, 15% B; 15 → 20 min, 85% → 10% A, 15% → 90% B. The flow rate was 1.0 mL/min, the injection volume was 10 μL, and the detection wavelength was 250 nm.

### Correlation analysis between the ecological factors and chemical components

In this study, ArcGIS software was used to determine the ecological factor values at the sampling sites for *A*. *membranaceus* var. *mongholicus.* Then, stepwise regression was performed using SPSS software (version 27.0) to conduct a correlation analysis of the chemical components of the medicinal materials, including plant protein, astragalus polysaccharide, astragaloside IV, calycosin-7-glucoside, dietary fibre, total flavonoids, and ecological factors. Finally, a regression model was constructed to assess the relationships between the chemical composition and ecological factors, while the spatial distribution of the chemical components of *A*. *membranaceus* var. *mongholicus* was estimated using ArcGIS software based on the regression model.

### Environmental variables

To identify the primary drivers of the distribution of *A*. *membranaceus* var. *mongholicus* in China, we incorporated 39 environmental variables, including 19 bioclimatic variables, seven soil variables, elevation, and solar radiation (Table 3). Data on the 19 bioclimatic variables, solar radiation, and elevation were obtained from the WorldClim database (https://www.worldclim.org), characterized by a 30 s spatial resolution and spanning the period from 1970 to 2000 [53]. The soil data were retrieved from the Harmonized World Soil Database (HWSD, http://www.fao.org/soils-portal/) [54]. For future projections, we utilize the emission scenarios of shared socioeconomic pathways (SSPs) and representative concentration pathways (RCPs) to drive the model. Precipitation and temperature projections were derived from three high-resolution general circulation models (MIROC5, BCC-CSM2-MR, and BCC-CSM1.1), with the climate change data for the MIROC5 and BCC-CSM1.1 models sourced from the Fifth Assessment Report (AR5) of the Intergovernmental Panel on Climate Change (IPCC). These models consider four distinct representative concentration pathways: RCP 2.6 (with a decreasing radiative level of 2.6 W/m^2^), RCP 4.5 (with a stabilizing radiative level of 4.5 W/m^2^), RCP 6.0 (with an intermediate radiative level of 6.0 W/m^2^) and RCP 8.5 (with an increase of 8.5 W/m^2^) [55]. In addition, BCC-CSM2-MR (National (Beijing) Climate Center Climate System Model) from the Coupled Model Intercomparison Project Phase 6 (CMIP6) was selected to project the conditions in China under four shared socioeconomic pathways (SSP126, SSP245, SSP370, and SSP585) [56]. These pathways represent radiative forcing levels of 2.6 W/m^2^ in 2100 (low-forcing scenario), 4.5 W/m^2^ in 2100 (medium-forcing scenario), 7.0 W/m^2^ in 2100 (medium- to high-forcing scenario), and 8.5 W/m^2^ in 2100 (high-forcing scenario) [57]. We projected the potential distribution of *A*. *membranaceus* var. *mongholicus* during two future periods, i.e., the 2050s and 2090s. To maintain consistency across the time series models, the soil and terrain variables remained unchanged for obtaining future predictions [58]. Therefore, in this study, we employed soil and terrain variables for the different periods in both the present and future analyses.

**Table 3:** Description of environmental variables used in Maxent

Abbreviation	Ecological factors	Unit
Bio1	Annual mean temperature	◦C
Bio2	Mean diurnal range (Mean of monthly)	◦C
Bio3	Isothermality	%
Bio4	Standard deviation of temperature seasonality	◦C
Bio5	Max temperature of warmest month	◦C
Bio6	Min temperature of coldest month	◦C
Bio7	Temperature annual range	◦C
Bio8	Mean temperature of wettest quarter	◦C
Bio9	Mean temperature of driest quarter	◦C
Bio10	Mean temperature of warmest quarter	◦C
Bio11	Mean temperature of coldest quarter	◦C
Bio12	Annual precipitation	mm
Bio13	Precipitation of wettest month	mm
Bio14	Precipitation of driest month	mm
Bio15	Precipitation seasonality (coefficient of variation)	mm
Bio16	Precipitation of wettest quarter	mm
Bio17	Precipitation of driest quarter	mm
Bio18	Precipitation of warmest quarter	mm
Bio19	Precipitation of coldest quarter	mm
Elev	Elevation	m
awc_class	AWC range	Code
t_ph_h2o	Topsoil pH (H_2_O)	− log (H +)
t_cec_soil	Topsoil CEC (soil)	cmol/kg
t_oc	Topsoil organic carbon	% weight
s_ph_h2o	Substrate-soil pH (H_2_O)	− log (H +)
s_cec_soil	Substrate-soil CEC (soil)	cmol/kg
s_oc	Substrate-soil organic carbon	% weight
Srad (01–12)	solar radiation	kJ m^−2^ day^−1^

**Fig. 7:**
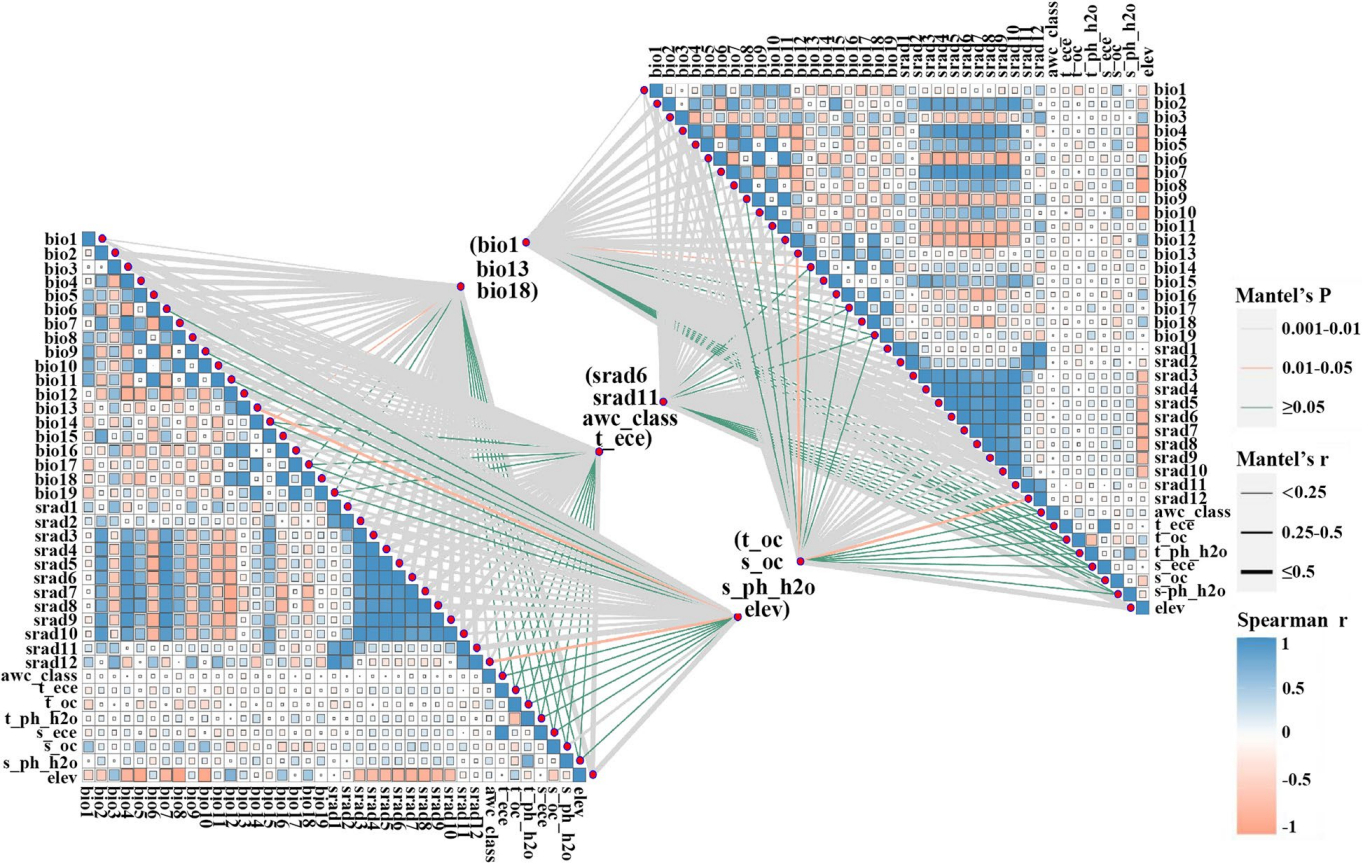
Migration of the center of suitable habitat for *A*.* membranaceus* var. *mongholicus *migratory routes in current and future climate scenarios. **a** Arrows indicate migratory routes and direction of the suitable habitat distribution center under current and future climate scenarios. **b** The bar chart represents the core distribution shift distance for *A*.* membranaceus* var. *mongholicus* under different scenarios/years. Among them, the meaning of the letters were (**A**) current, (**B**) SSP126-2050s, (**C**) SSP245-2050s, (**D**) SSP370-2050s, (**E**) SSP585-2050s, (**F**) SSP126-2090s, (**G**) SSP245-2090s, (**H**) SSP370-2090s, (**I**) SSP585-2090s

To prevent model overfitting due to the high correlation between the environmental variables [40, 59], it is necessary to screen the selected environmental factors. First, interpolation extraction of the environmental variable points was performed using ArcGIS 10.4. Subsequently, the interpolated data points were analysed using SPSS 27.0 through Spearman correlation and multicollinearity variance inflation factor (VIF) analysis. Environmental factors with Spearman correlation values less than 0.7 and VIF values less than 5 were selected for distribution prediction [10, 60, 61] (Fig. 7). The models included three climatic factors (Bio1, Bio13, and Bio18), five soil factors (Awc_class, T_ece, T_oc, S_oc, and S_ph_h2o), solar radiation, and one terrain factor (Table S3).

### Optimization models

In the construction of distribution models, the system provides a set of parameters by default, but these default parameters are influenced by the test data. With the default parameter settings, the sensitivity of the model to the test data increases, which may lead to overfitting, thus affecting the accuracy of the model prediction results. In this study, the Kuenm package in R was used to optimize the key parameters in the Maxent model, namely, the feature class (FC) and the regularization multiplier (RM) [62]. The RM value ranges from 0.1 to 4 and increases in steps of 0.1. The five FCs (linear L, quadratic Q, product P, threshold T, and hinge H) were permuted into 31 FC combinations. The fit and complexity of the parameter combinations were assessed using the Akaike Information criterion (AICc) [62, 63], and the ability of the different parameter combinations to differentiate test points from background points was evaluated by the area under the receiver operating characteristic curve [64]. In addition, based on the optimization results, we chose a model with an omission rate < 0.05 and a small-sample corrected delta Akaike information criterion ≤ 2 [65].

### Species distribution modelling process

The Maxent model is a Java-based artificial intelligence algorithm that integrates machine learning with statistical modelling to predict species distribution probabilities [66]. In this study, Maxent software version 3.4.1 (http://www.cs.princeton.edu/) was utilized alongside environmental variable datasets to analyse the distribution data of *A*. *membranaceus* var. *mongholicus*. We focused on modelling species distributions built using the current distribution data and current environmental variables [67] and subsequently modelled future distributions by entering 2050 or 2090 environmental data into the projection layer window [68]. In our modelling approach, 75% of the distribution points were randomly selected as the training dataset, while 25% of distribution points served as the test set. We used 100 repetitions in bootstrap mode and set the output format to logistic, with the other settings kept as default values (500 iterations, convergence threshold of 0.00001, and 10,000 background points) [69]. In this study, the predictive accuracy of the model was assessed using the area under the Receiver Operating Characteristic (ROC) curve and the true skill statistic (TSS) [70]. The AUC varies between 0 and 1, with values closer to 1 indicating enhanced predictive accuracy [71]. Specifically, an AUC value between 0.9 and 1 suggests an excellent model [72]. However, related studies have shown that the AUC values of the Maxent model need to be validated with the aid of constructing other related models to ensure that the model is valid. We used a null model to validate the accuracy of the Maxent model [73], and the results showed that the AUC value of the Maxent model was significantly larger than that of the null model, which indicated that the results of the model were accurate and valid (Fig S8 and Table S4). TSS value ranges from -1 to 1. A TSS value of 1 suggests that the model is nearly perfect, whereas values near or below 0 indicate that the model does not outperform a random model [70]. The model provides a continuous probability layer from 0 to 1, which enables the assessment of the suitability for plant growth in various natural environments and facilitates ecological suitable regionalization research. Drawing from the Maximum test sensitivity plus specificity [74], we categorized the suitability areas of *A*. *membranaceus* var. *mongholicus* into four levels: unsuitable (0.00–0.25), poorly suitable (0.25–0.50), moderately suitable (0.50–0.75), and highly suitable areas (> 0.75).

### Centroid migration in the core distribution

The Species Distribution Modeling (SDM) tool in ArcGIS was utilized for analysing the centroids of the suitable habitats for *A*. *membranaceus* var. *mongholicus* across present and future periods under the three climate change scenarios. This analysis aided in identifying the species’ migration directions and quantifying the centroid migration distances [75]. The specific methodology involved first converting the prediction results of the potentially suitable areas into binary vector data, where a species suitability probability of *p* ≥ 0.03 was considered the total suitable area, while *p* < 0.03 was considered to indicate unsuitable areas. Then, utilizing the zonal geometry feature within spatial analysis tools, we obtained the centroid coordinates of *A*. *membranaceus* var. *mongholicus* based on different climate projections. Finally, we categorized the suitability areas under the future climate conditions into three types: no change (areas that remain suitable both under the future and current climate conditions), contraction (areas that will disappear in the future compared to the those under the current climate conditions), and expansion (areas that will emerge as new in the future compared to those under the current climate conditions).

### Supplementary Information

Supplementary Material 1: Fig. S1 Spatial changes of *A*. *membranaceus* var. *mongholicus* in China under emission scenarios of the 2050s and 2090s. White, Gray, Red and Blue areas represent not suitable, unchanged suitable, expansion suitable, and contraction suitable areas, respectively. (a-d), the 2050s; (e–h), the 2090s; (a, e), future climate scenario SSP126; (b, f), future climate scenario SSP245; (c, g), future climate scenario SSP370; (d, h), future climate scenario SSP585. (Note: general circulation model BCC-CSM1.1). Fig. S2 Spatial changes of *A*. *membranaceus* var. *mongholicus* in China under emission scenarios of the 2050s and 2090s. White, Gray, Red and Blue areas represent not suitable, unchanged suitable, expansion suitable, and contraction suitable areas, respectively. (a-d), the 2050s; (e–h), the 2090s; (a, e), future climate scenario SSP126; (b, f), future climate scenario SSP245; (c, g), future climate scenario SSP370; (d, h), future climate scenario SSP585. (Note: general circulation model MIROC5). Fig. S3 Spatial changes of quality zonation in China under emission scenarios of the 2050s and 2090s. White, Gray, Red and Blue areas represent not suitable, unchanged suitable, expansion suitable, and contraction suitable areas, respectively. (a-d), the 2050s; (e–h), the 2090s; (a, e), future climate scenario SSP126; (b, f), future climate scenario SSP245; (c, g), future climate scenario SSP370; (d, h), future climate scenario SSP585. (Note: general circulation model BCC-CSM2-MR). Fig. S4 Spatial changes of quality zonation in China under emission scenarios of the 2050s and 2090s. White, Gray, Red and Blue areas represent not suitable, unchanged suitable, expansion suitable, and contraction suitable areas, respectively. (a-d), the 2050s; (e–h), the 2090s; (a, e), future climate scenario SSP126; (b, f), future climate scenario SSP245; (c, g), future climate scenario SSP370; (d, h), future climate scenario SSP585. (Note: general circulation model BCC-CSM1.1). Fig. S5 Spatial changes of quality zonation in China under emission scenarios of the 2050s and 2090s. White, Gray, Red and Blue areas represent not suitable, unchanged suitable, expansion suitable, and contraction suitable areas, respectively. (a-d), the 2050s; (e–h), the 2090s; (a, e), future climate scenario SSP126; (b, f), future climate scenario SSP245; (c, g), future climate scenario SSP370; (d, h), future climate scenario SSP585. (Note: general circulation model MIROC5). Fig. S6 Migration of the center of suitable habitat for quality zonation migratory routes in current and future climate scenarios. (a) Arrows indicate migratory routes and direction of the suitable habitat distribution center under current and future climate scenarios. (b) The bar chart represents the core distribution shift distance for quality zonation under different scenarios/years. Among them, the meaning of the letters were (A) current, (B) SSP126-2050s, (C) SSP245-2050s, (D) SSP370-2050s, (E) SSP585-2050s, (F) SSP126-2090s, (G) SSP245-2090s, (H) SSP370-2090s, (I) SSP585-2090s. Fig. S7 Response curves of the current existence probability of *A*. *membranaceus* var. *mongholicus* to the bioclimatic variables. Fig. S8 Statistical analysis of null model AUC values.

## Data Availability

All relevant data can be found within the manuscript.
