# Supplementary material for: Evaluation of the environmental factors influencing the quality of Astragalus membranaceus var. mongholicus based on HPLC and the Maxent model
Source: BMC Plant Biol. 2024 Jul 23;24:697. doi: 10.1186/s12870-024-05355-3 (PMC11264576; doi:10.1186/s12870-024-05355-3)
Supplement: Supplementary file 1 — Supplementary Material 1: Fig. S1 Spatial changes of A. membranaceus var. mongholicus in China under emission scenarios of the 2050s and 2090s. White, Gray, Red and Blue areas represent not suitable, unchanged suitable, expansion suitable, and contraction suitable areas, respectively. (a-d), the 2050s; (e–h), the 2090s; (a, e), future climate scenario SSP126; (b, f), future climate scenario SSP245; (c, g), future climate scenario SSP370; (d, h), future climate scenario SSP585. (Note: general circulation model BCC-CSM1.1). Fig. S2 Spatial changes of A. membranaceus var. mongholicus in China under emission scenarios of the 2050s and 2090s. White, Gray, Red and Blue areas represent not suitable, unchanged suitable, expansion suitable, and contraction suitable areas, respectively. (a-d), the 2050s; (e–h), the 2090s; (a, e), future climate scenario SSP126; (b, f), future climate scenario SSP245; (c, g), future climate scenario SSP370; (d, h), future climate scenario SSP585. (Note: general circulation model MIROC5). Fig. S3 Spatial changes of quality zonation in China under emission scenarios of the 2050s and 2090s. White, Gray, Red and Blue areas represent not suitable, unchanged suitable, expansion suitable, and contraction suitable areas, respectively. (a-d), the 2050s; (e–h), the 2090s; (a, e), future climate scenario SSP126; (b, f), future climate scenario SSP245; (c, g), future climate scenario SSP370; (d, h), future climate scenario SSP585. (Note: general circulation model BCC-CSM2-MR). Fig. S4 Spatial changes of quality zonation in China under emission scenarios of the 2050s and 2090s. White, Gray, Red and Blue areas represent not suitable, unchanged suitable, expansion suitable, and contraction suitable areas, respectively. (a-d), the 2050s; (e–h), the 2090s; (a, e), future climate scenario SSP126; (b, f), future climate scenario SSP245; (c, g), future climate scenario SSP370; (d, h), future climate scenario SSP585. (Note: general circulation model BCC-CSM1.1). F [file 12870_2024_5355_MOESM1_ESM.zip › Table S1.docx]

**Table S1** Contents (mg/g) of the six Chemical composition in rhizoma of *A*. *membranaceus* var. *mongholicus* herbs determined by the HPLC method.

| **Sample No.** | **Geographical origins** | **Plant protein** | **Astragaloside IV** | **Calycosin-7-glucoside** | **Astragalus polysaccharide** | **Dietary fiber** | **Total flavonoids** |
| --- | --- | --- | --- | --- | --- | --- | --- |
| 1 | Shuikeng Township, Yanchi County, Ningxia | 7.50 | 0.227 | 0.028 | 14.90 | 42.87 | 134 |
| 2 | Chenjin Township, Lunde County, Guyuan City, Ningxia | / | 0.381 | 0.024 | / | / | / |
| 3 | Chenjin Township, Lunde County, Guyuan City, Ningxia | 7.27 | 0.209 | 0.049 | 12.11 | 45.89 | 55.5 |
| 4 | Baiyang Town, Guyuan City, Ningxia | / | 0.223 | 0.037 | / | / | / |
| 5 | Yongan Township, Hunyuan County, Datong City, Shanxi | 7.67 | 0.146 | 0.042 | 16.43 | 41.82 | 184 |
| 6 | Hunyuan County, Datong City, Shanxi | / | 0.236 | 0.104 | / | / | / |
| 7 | Shirenshan Village, Zhaizhuang Township, Hunyuan County, Datong City, Shanxi | / | 0.259 | 0.097 | / | / | / |
| 8 | Mazhuang Village, Guan'er Township, Hunyuan County, Shanxi | / | 0.223 | 0.074 | / | / | / |
| 9 | Linchang Village, Darenzhuang Township, Hunyuan County, Shanxi | / | 0.201 | 0.073 | / | / | / |
| 10 | Heituwan Village, Baimashi Township, Ying County, Shuozhou City, Shanxi | / | 0.257 | 0.078 | / | / | / |
| 11 | Shanqiao Village, Baimashi Township, Ying County, Shuozhou City, Shanxi | / | 0.197 | 0.068 |  |  |  |
| 12 | Yue Yanzhuang Village, Daying Township, Fanchi County, Shuozhou City, Shanxi | / | 0.132 | 0.082 | / | / | / |
| 13 | Huai Ning Wan Township, Zizhou County, Yulin City, Shaanxi | / | 0.228 | 0.035 | / | / | / |
| 14 | Zizhou County, Yulin City, Shaanxi | 5.21 | 0.228 | 0.046 | 11.40 | 51.60 | 485 |
| 15 | Mizhi County, Yulin City, Shaanxi | / | 0.240 | 0.023 | / | / | / |
| 16 | Inner Mongolia University, Hohhot City, Inner Mongolia | 6.66 | 0.184 | 0.052 | 12.22 | 40.96 | 273 |
| 17 | Deshenggou Township, Wuchuan County, Hohhot City, Inner Mongolia | / | 0. 087 | 0.054 | / | / | / |
| 18 | Wuchuan County, Hohhot City, Inner Mongolia | / | 0.085 | 0.068 | / | / | / |
| 19 | Xiashitengou Township, Guyang County, Baotou City, Inner Mongolia | / | 0. 158 | 0.090 | / | / | / |
| 20 | Huishuo Town, Guyang County, Baotou City, Inner Mongolia | / | 0.121 | 0.048 | / | / | / |
| 21 | Xingshunxi Township, Guyang County, Baotou City, Inner Mongolia, China | / | 0.125 | 0.092 | / | / | / |
| 22 | Chifeng City, Inner Mongolia | / | 0.359 | 0.077 | / | / | / |
| 23 | Chaoyang Township, Ulatqian Banner, Bayannur City, Inner Mongolia | / | 0.136 | 0.081 | / | / | / |
| 24 | Chifeng City, Inner Mongolia | / | 0.126 | 0.031 | / | / |  |
| 25 | Longxi County, Dingxi City, Gansu | 8.93 | 0.284 | 0.052 | 22.10 | 37.64 | 201 |
| 26 | Longxi County, Dingxi City, Gansu | 7.11 | 0.288 | 0.057 | 11.50 | 41.39 | 176 |
| 27 | Zhangjiawan, Longxi County, Dingxi City, Gansu | 8.53 | 0.276 | 0.032 | 9.51 | 38.11 | 121 |
| 28 | Xueshan Village, Longxi County, Dingxi City, Gansu | / | 0.348 | 0.084 | / | / | / |
| 29 | Dongjiabao, Longxi County, Dingxi City, Gansu | 6.29 | 0.214 | 0.095 | 9.46 | 46.39 | 272 |
| 30 | Longxi County, Dingxi City, Gansu | 8.08 | 0.240 | 0.046 | 10.31 | 37.84 | 182 |
| 31 | Shixiawan Township, Dingxi City, Gansu | / | 0.234 | 0.034 | / | / | / |
| 32 | Longshan Town, Tongwei County, Dingxi City, Gansu | / | 0.266 | 0.037 | / | / | / |
| 33 | Weiyuan County, Dingxi City, Gansu | 6.98 | 0.246 | 0.082 | 8.86 | 43.29 | 213 |
| 34 | Lintao County, Dingxi City, Gansu | 8.04 | 0.229 | 0.051 | 4.62 | 47.68 | 152 |
| 35 | Minxian, Dingxi City, Gansu | 7.91 | 0.194 | 0.047 | 11.50 | 30.73 | 299 |
| 36 | Minxian, Dingxi City, Gansu | / | 0.195 | 0.060 | / | / | / |
| 37 | Dangchang County, Gansu | 7.90 | 0.174 | 0.053 | 7.31 | 38.59 | 144 |
| 38 | Zhang County, Dingxi City, Gansu | 7.87 | 0.272 | 0.044 | 21.86 | 42.12 | 175 |
| 39 | Weiyuan County, Dingxi City, Gansu | / | 0.223 | 0.084 | / | / | / |
