# Supplementary material for: Evaluation of the environmental factors influencing the quality of Astragalus membranaceus var. mongholicus based on HPLC and the Maxent model
Source: BMC Plant Biol. 2024 Jul 23;24:697. doi: 10.1186/s12870-024-05355-3 (PMC11264576; doi:10.1186/s12870-024-05355-3)
Supplement: Supplementary file 1 — Supplementary Material 1: Fig. S1 Spatial changes of A. membranaceus var. mongholicus in China under emission scenarios of the 2050s and 2090s. White, Gray, Red and Blue areas represent not suitable, unchanged suitable, expansion suitable, and contraction suitable areas, respectively. (a-d), the 2050s; (e–h), the 2090s; (a, e), future climate scenario SSP126; (b, f), future climate scenario SSP245; (c, g), future climate scenario SSP370; (d, h), future climate scenario SSP585. (Note: general circulation model BCC-CSM1.1). Fig. S2 Spatial changes of A. membranaceus var. mongholicus in China under emission scenarios of the 2050s and 2090s. White, Gray, Red and Blue areas represent not suitable, unchanged suitable, expansion suitable, and contraction suitable areas, respectively. (a-d), the 2050s; (e–h), the 2090s; (a, e), future climate scenario SSP126; (b, f), future climate scenario SSP245; (c, g), future climate scenario SSP370; (d, h), future climate scenario SSP585. (Note: general circulation model MIROC5). Fig. S3 Spatial changes of quality zonation in China under emission scenarios of the 2050s and 2090s. White, Gray, Red and Blue areas represent not suitable, unchanged suitable, expansion suitable, and contraction suitable areas, respectively. (a-d), the 2050s; (e–h), the 2090s; (a, e), future climate scenario SSP126; (b, f), future climate scenario SSP245; (c, g), future climate scenario SSP370; (d, h), future climate scenario SSP585. (Note: general circulation model BCC-CSM2-MR). Fig. S4 Spatial changes of quality zonation in China under emission scenarios of the 2050s and 2090s. White, Gray, Red and Blue areas represent not suitable, unchanged suitable, expansion suitable, and contraction suitable areas, respectively. (a-d), the 2050s; (e–h), the 2090s; (a, e), future climate scenario SSP126; (b, f), future climate scenario SSP245; (c, g), future climate scenario SSP370; (d, h), future climate scenario SSP585. (Note: general circulation model BCC-CSM1.1). F [file 12870_2024_5355_MOESM1_ESM.zip › Table S2.docx]

| **Species** | **Model** | **Period** | **Area of each suitable region (× 10^4^ Km^2^)** | | | |
| --- | --- | --- | --- | --- | --- | --- |
|  |  |  | **Unsuitable region** | **Unchanged region** | **Expansion region** | **Contractionregion** |
| *Astragalus membranaceus* var. *mongholicus* | BCC-CSM2-MR | Present **vs** SSP126-2050 | 631.89 | 15.28 | 10.46 | 10.82 |
|  |  | Present **vs** SSP245-2050s | 631.91 | 15.31 | 10.44 | 10.55 |
|  |  | Present **vs** SSP370-2050s | 635.05 | 15.21 | 7.30 | 11.53 |
|  |  | Present **vs** SSP585-2050s | 634.59 | 14.93 | 7.76 | 14.38 |
|  |  | Present **vs** SSP126-2090s | 629.76 | 15.52 | 12.59 | 8.45 |
|  |  | Present **vs** SSP245-2090s | 635.33 | 15.23 | 8.03 | 8.71 |
|  |  | Present **vs** SSP370-2090s | 633.56 | 15.19 | 8.80 | 11.64 |
|  |  | Present **vs** SSP585-2090s | 634.32 | 15.49 | 7.02 | 11.36 |
|  | BCC-CSM1.1 | Present **vs** RCP2.6-2050s | 630.42 | 15.25 | 11.86 | 11.17 |
|  |  | Present **vs** RCP4.5-2050s | 629.36 | 15.34 | 12.91 | 10.21 |
|  |  | Present **vs** RCP6.0-2050s | 631.42 | 15.31 | 10.85 | 10.57 |
|  |  | Present vs RCP8.5-2050s | 632.75 | 14.92 | 9.52 | 14.46 |
|  |  | Present vs RCP2.6-2090s | 631.56 | 15.05 | 11.69 | 9.75 |
|  |  | Present vs RCP4.5-2090s | 630.58 | 15.39 | 10.72 | 13.09 |
|  |  | Present vs RCP6.0-2090s | 631.23 | 15.21 | 11.04 | 11.58 |
|  |  | Present vs RCP8.5-2090s | 630.67 | 15.15 | 11.61 | 12.17 |
|  | MIROC5 | Present **vs** RCP2.6-2050s | 633.68 | 14.89 | 8.59 | 14.72 |
|  |  | Present **vs** RCP4.5-2050s | 635.54 | 14.79 | 6.73 | 15.75 |
|  |  | Present **vs** RCP6.0-2050s | 634.85 | 14.93 | 7.42 | 14.34 |
|  |  | Present vs RCP8.5-2050s | 632.37 | 14.43 | 9.90 | 19.38 |
|  |  | Present vs RCP2.6-2090s | 632.13 | 14.65 | 10.14 | 17.10 |
|  |  | Present vs RCP4.5-2090s | 633.89 | 14.88 | 8.39 | 14.85 |
|  |  | Present vs RCP6.0-2090s | 632.77 | 14.79 | 9.50 | 15.74 |
|  |  | Present vs RCP8.5-2090s | 630.65 | 14.92 | 11.62 | 14.44 |

**Table S2** The potential distribution area of quality zonation in the 2050s and 2090s.
