# Supplementary material for: Evaluation of the environmental factors influencing the quality of Astragalus membranaceus var. mongholicus based on HPLC and the Maxent model
Source: BMC Plant Biol. 2024 Jul 23;24:697. doi: 10.1186/s12870-024-05355-3 (PMC11264576; doi:10.1186/s12870-024-05355-3)
Supplement: Supplementary file 1 — Supplementary Material 1: Fig. S1 Spatial changes of A. membranaceus var. mongholicus in China under emission scenarios of the 2050s and 2090s. White, Gray, Red and Blue areas represent not suitable, unchanged suitable, expansion suitable, and contraction suitable areas, respectively. (a-d), the 2050s; (e–h), the 2090s; (a, e), future climate scenario SSP126; (b, f), future climate scenario SSP245; (c, g), future climate scenario SSP370; (d, h), future climate scenario SSP585. (Note: general circulation model BCC-CSM1.1). Fig. S2 Spatial changes of A. membranaceus var. mongholicus in China under emission scenarios of the 2050s and 2090s. White, Gray, Red and Blue areas represent not suitable, unchanged suitable, expansion suitable, and contraction suitable areas, respectively. (a-d), the 2050s; (e–h), the 2090s; (a, e), future climate scenario SSP126; (b, f), future climate scenario SSP245; (c, g), future climate scenario SSP370; (d, h), future climate scenario SSP585. (Note: general circulation model MIROC5). Fig. S3 Spatial changes of quality zonation in China under emission scenarios of the 2050s and 2090s. White, Gray, Red and Blue areas represent not suitable, unchanged suitable, expansion suitable, and contraction suitable areas, respectively. (a-d), the 2050s; (e–h), the 2090s; (a, e), future climate scenario SSP126; (b, f), future climate scenario SSP245; (c, g), future climate scenario SSP370; (d, h), future climate scenario SSP585. (Note: general circulation model BCC-CSM2-MR). Fig. S4 Spatial changes of quality zonation in China under emission scenarios of the 2050s and 2090s. White, Gray, Red and Blue areas represent not suitable, unchanged suitable, expansion suitable, and contraction suitable areas, respectively. (a-d), the 2050s; (e–h), the 2090s; (a, e), future climate scenario SSP126; (b, f), future climate scenario SSP245; (c, g), future climate scenario SSP370; (d, h), future climate scenario SSP585. (Note: general circulation model BCC-CSM1.1). F [file 12870_2024_5355_MOESM1_ESM.zip › Table S3.docx]

**Table S3** The contribution rate for main ecological factors of *A*. *membranaceus* var. *mongholicus*

| **Type** | **Variables** | **Description** | **Contribution (%)** | **Permutation importance (%)** | **UNITS** |
| --- | --- | --- | --- | --- | --- |
| *A.* *membranaceus* var. *mongholicus* | Bio1 | Annual mean temperature | 5.5 | 21.2 | ℃ |
|  | Bio13 | Precipitation of wettest month | 14.3 | 23.5 | mm |
|  | Bio 18 | Precipitation of warmest quarter | 12.5 | 23 | mm |
|  | Srad6 | Solar radiation in June | 17.5 | 6.3 | kJ m^-2^ day^-1^ |
|  | Srad11 | Solar radiation in November | 17.6 | 11.3 | kJ m^-2^ day^-1^ |
|  | Awc_class | AWC range | 2.3 | 3.8 | Code |
|  | T_ece | Topsoil CEC (soil) | 0.2 | 0.3 | cmol/kg |
|  | T_oc | Topsoil organic carbon | 0.8 | 1.2 | % weight |
|  | S_oc | Substrate-soil organic carbon | 0.3 | 0.2 | % weight |
|  | S_ph_h2o | Substrate-soil pH (H_2_O) | 0.7 | 0.9 | −log (H+) |
|  | Elev | Elevation | 27.5 | 8.3 | m |
| Astragaloside IV | Bio12 | Annual precipitation | 24.2 | 41.3 | mm |
|  | Srad6 | Solar radiation in June | 17.6 | 7.1 | kJ m^-2^ day^-1^ |
|  | Bio11 | Mean temperature of coldest quarter | 4.5 | 8.9 | ◦C |
|  | Bio2 | Mean diurnal range (Mean of monthly) | 0.5 | 1.2 | ◦C |
|  | Elev | Elevation | 34.1 | 25.1 | m |
| Astragalus polysaccharides | Bio1 | Annual mean temperature | 26.7 | 10 | ℃ |
|  | Bio18 | Precipitation of warmest quarter | 15.5 | 11.7 | mm |
|  | Bio13 | Precipitation of wettest month | 18.3 | 29.7 | mm |
|  | Bio6 | Min temperature of coldest month | 15.9 | 10.5 | ℃ |
|  | Bio10 | Mean temperature of warmest quarter | 21.3 | 16.6 | ℃ |
|  | Bio16 | Precipitation of wettest quarter | 2.4 | 25 | mm |
